# Effect of dietary formic acid and astaxanthin on the survival and growth of Pacific white shrimp (*Litopenaeus vannamei*) and their resistance to *Vibrio parahaemolyticus*

**DOI:** 10.1186/s40064-015-1234-x

**Published:** 2015-08-21

**Authors:** Niti Chuchird, Phitsanu Rorkwiree, Tirawat Rairat

**Affiliations:** Faculty of Fisheries, Aquaculture Business Research Center, Kasetsart University, Bangkok, 10900 Thailand

**Keywords:** Astaxanthin, Formic acid, Pacific white shrimp, *Vibrio parahaemolyticus*

## Abstract

A 90-day feeding trial was conducted to evaluate the effects of formic acid (FA) and astaxanthin (AX) on growth, survival, immune parameters, and tolerance to *Vibrio* infection in Pacific white shrimp. The study was divided into two experiments. In experiment 1, postlarvae-12 were randomly distributed into six groups and then fed four times daily with six experimental diets contained 0.3 % FA, 0.6 % FA, 50 ppm AX, 0.3 % FA + 50 ppm AX, 0.6 % FA + 50 ppm AX, or none of these supplements (control diet). After 60 days of the feeding trials, the body weight of all treatment groups was not significantly different from the control group, although shrimp fed formic acid had significantly lower body weight than shrimp fed 50 ppm AX. However, the 0.6 % FA + 50 ppm AX group had a significantly higher survival rate (82.33 ± 8.32 %) than the control group (64.33 ± 10.12 %). In experiment 2, *Vibrio parahaemolyticus* was added to each tank to obtain a final concentration of 10^4^ colony-forming units/mL. Each treatment group received the aforementioned diets for another 30 days. At the end of this experiment, there was no difference in the weight gain among all experimental groups. However, the survival rate of shrimps whose diet included FA, AX, and their combination (in the range of 45.83–67.50 %) was significantly higher than the control group (20.00 ± 17.32 %). FA-fed shrimps also had significantly lower total intestinal bacteria and *Vibrio* spp. counts, while immune parameters [total hemocyte count (THC), phagocytosis activity, phenoloxidase (PO) activity, and superoxide dismutase (SOD) activity] of AX-fed groups were significantly improved compared with the other groups. In conclusion, FA, AX, and their combination are useful in shrimp aquaculture.

**Electronic supplementary material:**

The online version of this article (doi:10.1186/s40064-015-1234-x) contains supplementary material, which is available to authorized users.

## Background

Pacific white shrimp (*Litopenaeus vannamei*) is the primary shrimp species cultured in many Asian countries, including Thailand (Limsuwan and Chanratchakool 2004). Since 2012, Thai shrimp farmers have suffered major economic losses owing to Early Mortality Syndrome (EMS). The disease reduced shrimp production of Thailand from 5,40,000 tons in 2012 to 2,56,000 tons in 2013 and 2,10,000 tons in 2014, respectively (Office of Agricultural Economics 2014; Tangtong 2015). Affected shrimp show signs of a pale coloration owing to pigment loss, as well as an atrophied hepatopancreas. These signs may become apparent as early as 4 days after stocking (Munkongwongsiri et al. 2013). *Vibrio parahaemolyticus* is the suspected agent that causes mass mortality as it induced 100 % mortality with typical EMS pathology to experimental shrimp (Tran et al. 2013). Because the usage of antibiotics in shrimp aquaculture is discouraged, it is necessary to find an alternative solution to prevent bacterial infection. Organic acids are among the most promising substances as they have been reported to possess anti-*Vibrio* spp. activities (Mine and Boopathy 2011; Adams and Boopathy 2013; da Silva et al. 2013), and increased survival rate of shrimps (Walla et al. 2012; Su et al. 2014; Romano et al. 2015; Ng et al. 2015). Astaxanthin, a type of carotenoid, can also improve shrimp survival rate and enhance resistance to several stress conditions, such as low dissolved oxygen, low salinity, low temperature, and ammonia stress (Chien et al. 2003; Pan et al. 2003; Chien and Shiau 2005; Flores et al. 2007; Niu et al. 2009). Therefore, both organic acids and astaxanthin have the potential to be used in shrimp farming as feed additives. The objectives of this study were to evaluate the effect of dietary supplementation of formic acid and astaxanthin on growth, survival and tolerance to *V. parahaemolyticus* infection in Pacific white shrimp under laboratory conditions. The effects of these substances on total intestinal bacterial counts, intestinal *Vibrio* spp. counts, and some immune parameters of shrimp were also examined.

## Methods

*Experiment 1* The effects of formic acid and astaxanthin on growth and survival of Pacific white shrimp postlarvae.

### Experimental diets

Formic acid (FA) and astaxanthin (AX) used in this study were Amasil^®^ NA (94 % formic acid, BASF The Chemical Company, Germany) and Lucantin Pink^®^ CWD (10 % astaxanthin, BASF The Chemical Company, Germany). Six experimental diets were formulated; 0.3 % FA, 0.6 % FA, 50 ppm AX, 0.3 % FA + 50 ppm AX, 0.6 % FA + 50 ppm AX, or none of these supplements (control diet). Both substances were applied by spraying and mixing with commercial pellet feed containing 36 % crude protein and 6 % lipid from Charoen Pokphand, Thailand.

### Shrimps and experimental protocol

The experiments were carried out at the Aquaculture Business Research Center Laboratory, Faculty of Fisheries, Kasetsart University, Thailand. Postlarvae-9 (PL-9) of Pacific white shrimp were obtained from a hatchery in Chachoengsao Province, Thailand. After 3 days of acclimation, shrimps (PL-12) were randomly distributed into 24 × 500-L fiberglass tanks (four replicate tanks per treatment). Each tank was stocked with 75 shrimp. Each treatment group was fed with one of the six diets four times daily to satiation for 60 days. Salinity throughout the experiment was maintained at 25 ppt, dissolved oxygen above 4 ppm, and water temperature at 29 ± 1 °C. Leftover feed and feces were siphoned daily, and 10 % of the water was exchanged every 3 days. The average body weight and survival rate of shrimp were recorded after a 60-day experimental period. Ten shrimps from each tank were randomized and weighted individually by two-decimal point balance.

*Experiment 2* The effects of formic acid and astaxanthin on growth, survival, intestinal bacteria, and immune responses of Pacific white shrimps challenged with *Vibrio parahaemolyticus*.

### Shrimps and experimental protocol

Shrimps from each tank in experiment 1 were randomly distributed into new 24 × 500-L fiberglass tanks (four replicate tanks per treatment). The stocking density was 30 shrimps per tank. At the beginning of this experiment (0 day), *Vibrio parahaemolyticus* was added into each tank to obtain final concentration of 10^4^ colony-forming units (CFU)/mL, which is the normal concentration of *Vibrio* in the water of shrimp farm as described by Sung et al. (2001) and Lavilla-Pitogo et al. (1998). *V. parahaemolyticus* used for immersion challenge test in this study was collected from the EMS farm in Thailand using method described by Joshi et al. (2014). Each treatment group received the same diet as in experiment 1 four times daily for another 30 days. Salinity, dissolved oxygen, and water temperature were maintained as in the experiment 1. Leftover feed and feces were siphoned every 2 days.

### Growth and survival study

The weight of shrimp from each treatment was measured and their survival rate was recorded on the 30th day after being challenged with *V. parahaemolyticus* at 10^4^ CFU/mL.

### Intestinal bacterial study

Five shrimp from each group were randomized and their intestines collected on the 10th, 20th, and 30th day. The intestine of each shrimp was homogenized and spread on TCBS (selective media for *Vibrio* spp. culture) or NA (general media for most bacterial cultures) by the spread plate technique, then incubated at 37 °C for 24 h. Finally, all colonies of bacteria were counted and calculated as CFU/g unit.

### Immune parameters study

The immune parameters were measured at the end of the feeding trial. Ten shrimp per treatment were used for immunological tests. A hemolymph sample of 250 µL from each shrimp was withdrawn from the base of the 3rd walking leg using a syringe containing 750 µL of precooled (4 °C) anticoagulant (0.114 M trisodium citrate, 450 mM NaCl, 10 mM KCl, 10 mM HEPES at pH 7.4) (Nonwachai et al. 2010). The hemolymph-anticoagulant mixture was used to measure total hemocyte count (THC), phagocytosis activity, phenoloxidase (PO) activity, superoxide dismutase (SOD) activity, and bactericidal activity.

1. Total hemocyte count
  After collecting hemolymph, hemocytes were counted using a hemocytometer and calculated as THC (cells/mL) = count × 10^4^ × dilution factor.
2. Phagocytosis activity
  Phagocytotic activity was determined according to Itami et al. (1994). Collected shrimp hemocytes were rinsed with shrimp saline (a solution of NaCl 28.4 g, MgCl_2_·6H_2O_ 1.0 g, MgSO4·7H_2O_ 2.0 g, CaCl_2_·2H_2O_ 2.25 g, KCl 0.7 g, glucose 1.0 g, and HEPES 2.38 g/L) and the viable cell number adjusted to 1 × 10^6^ cells/mL. The cell suspension (200 µL) was inoculated onto a cover slip. After 20 min, the cell suspension was removed and rinsed with shrimp saline three times. Heat-killed yeast preparation (2 mL) was added and incubated for 2 h. Next, the heat-killed yeast preparation was removed and the cell suspension rinsed with shrimp saline five times to reach a concentration of 5 × 10^8^ cells/mL, and fixed with 100 % methanol. Then, the cover slip was stained with Giemsa stain and mounted with Permount slide mounting fluid. Two hundred hemocytes were counted for each sample. Phagocytic activity, defined as percentage phagocytosis was expressed as:
  $${\text{Percentage phagocytosis}} = ({\text{phagocytic hemocytes}}/{\text{total hemocytes}}) \times 100$$
3. Phenoloxidase activity
  Phenoloxidase activity was measured spectrophotometrically by recording the formation of dopachrome produced from l-dihydroxyphenylalanine, following a modification of a published protocol (Supamattaya et al. 2000). The hemolymph-anticoagulant mixture was washed three times with shrimp saline and centrifuged at 1000 rpm and 4 °C for 10 min. Hemocyte lysate was prepared from hemocytes in cacodylate buffer (pH 7.4; 0.01 M sodium cacodylate, 0.45 M sodium chloride, 0.01 M calcium chloride, and 0.26 M magnesium chloride; pH 7.0) by using a sonicator at 30 amplitude for 5 s, and the suspension was then centrifuged at 10,000 rpm at 4 °C for 20 min and the supernatant collected. Then 200 µL of 0.25 % trypsin in cacodylate buffer was mixed into the 200 µL of hemocyte lysate followed by 200 µL of l-dihydroxyphenylalanine at 4 mg/mL as substrate. Enzyme activity was measured as the absorbance of dopachrome at 490 nm wavelength. The protein content in hemocyte lysate was measured following a published protocol (Lowry et al. 1951). The phenoloxidase activity was calculated as the increase in optimum density per minute per milligram of protein.
4. Superoxide dismutase activity
  SOD activity was measured by its ability to inhibit superoxide radical-dependent reactions using a Ransod Kit (Randox, Crumlin, UK). This method is based on the formation of red formazan during a reaction of 2-(4-iodophenyl)-3-(4-nitrophenol)-5-phenyltetrazolium chloride (INT) and superoxide radical, which is assayed in a spectrophotometer at 505 nm. The reaction mixture (1.7 mL) contains 0.05 mM xanthine and 0.025 mM INT dissolved in 50 mM CAPS (pH 10.2) and 0.94 mM EDTA. In the presence of xanthine oxidase, superoxide and uric acid are produced from the xanthine. The superoxide radicals then react with INT to produce a red formazan dye. The hemolymph-anticoagulant mixture was centrifuged at 3000 rpm and 4 °C for 10 min. Plasma was removed, and the pellet was resuspended with 3 mL of 0.9 % NaCl and centrifuged again. The supernatant was discarded, and the pellet was resuspended with 2 mL of triple distilled water at 4 °C. A 50 µL aliquot of resuspended hemocytes was placed in each well of a 96-well plate that contained 200 µL of reaction mixture. Fifty microliters of xanthine oxidase solution was added to each well, and the absorbance measured at 505 nm and 37 °C. The rate of reaction was estimated from the absorbance readings of 0.5 and 3 min after adding xanthine oxidase. A reference standard of SOD was supplied with the Ransod Kit. One unit of SOD was defined as the amount required to inhibit the rate of xanthine reduction by 50 %. The specific activity was expressed as SOD units/mL.
5. Bactericidal activity
  Bactericidal activity was measured as described by Supamattaya et al. (2000). Serum was separated from the hemocytes of each shrimp sample before diluting in 2.6 % NaCl at the following ratios: 1:2, 1:4, 1:8, 1:16, and 1:32. Then 0.5 mL of each serum dilution was used for the assay. For the negative control, 0.1 mL of NaCl was used in the assay. One tenth of a milliliter of *Vibrio harveyi* suspension (8.2 × 10^6^ CFU/mL) was added to each serum dilution and the control. The treatments were incubated at room temperature for 3 h before enumerating the bacteria. The results were recorded from a dilution that could decrease 50 % of *V. harveyi* compared with the control.

### Statistical analysis

Results are presented as the mean ± standard deviation. One way ANOVA and Duncan’s New Multiple Range test were used to compare data among treatments. Differences were considered significant if p < 0.05.

## Results

*Experiment 1* The effects of formic acid and astaxanthin on growth and survival of Pacific white shrimp postlarvae

After 60 days of dietary administration, shrimp fed with 50 ppm AX had the highest average body weight (4.45 ± 0.45 g), followed by shrimp fed with 0.3 % FA + 50 ppm AX (4.38 ± 0.37 g), 0.6 % FA + 50 ppm AX (4.05 ± 0.21 g) and the control group (4.18 ± 0.05 g). However, the body weight of all FA and AX-fed shrimps were not significantly different from the control group. The average survival rate of shrimp fed with 0.6 % FA + 50 ppm AX was 82.33 ± 8.32 % which was highest among all the other groups and significantly higher than the control group (64.33 ± 10.12 %) (Additional file 1: Table S1).

*Experiment 2* The effects of formic acid and astaxanthin on growth, survival, intestinal bacteria, and immune responses of Pacific white shrimps challenged with *Vibrio parahaemolyticus.*

At the end of the feeding trial, the average weight gain of 0.3 % FA + 50 ppm AX-fed shrimp was highest, being 2.97 ± 0.83 g. Nevertheless, no significant differences among the six experimental groups were observed. The average survival rate of all FA and AX-fed shrimps were significantly higher than the control group (20.00 ± 17.32 %) and the best result was obtained in the 0.6 % FA + 50 ppm AX-fed group (67.50 ± 3.33 %) (Additional file 1: Table S2).

**Fig. 1:**
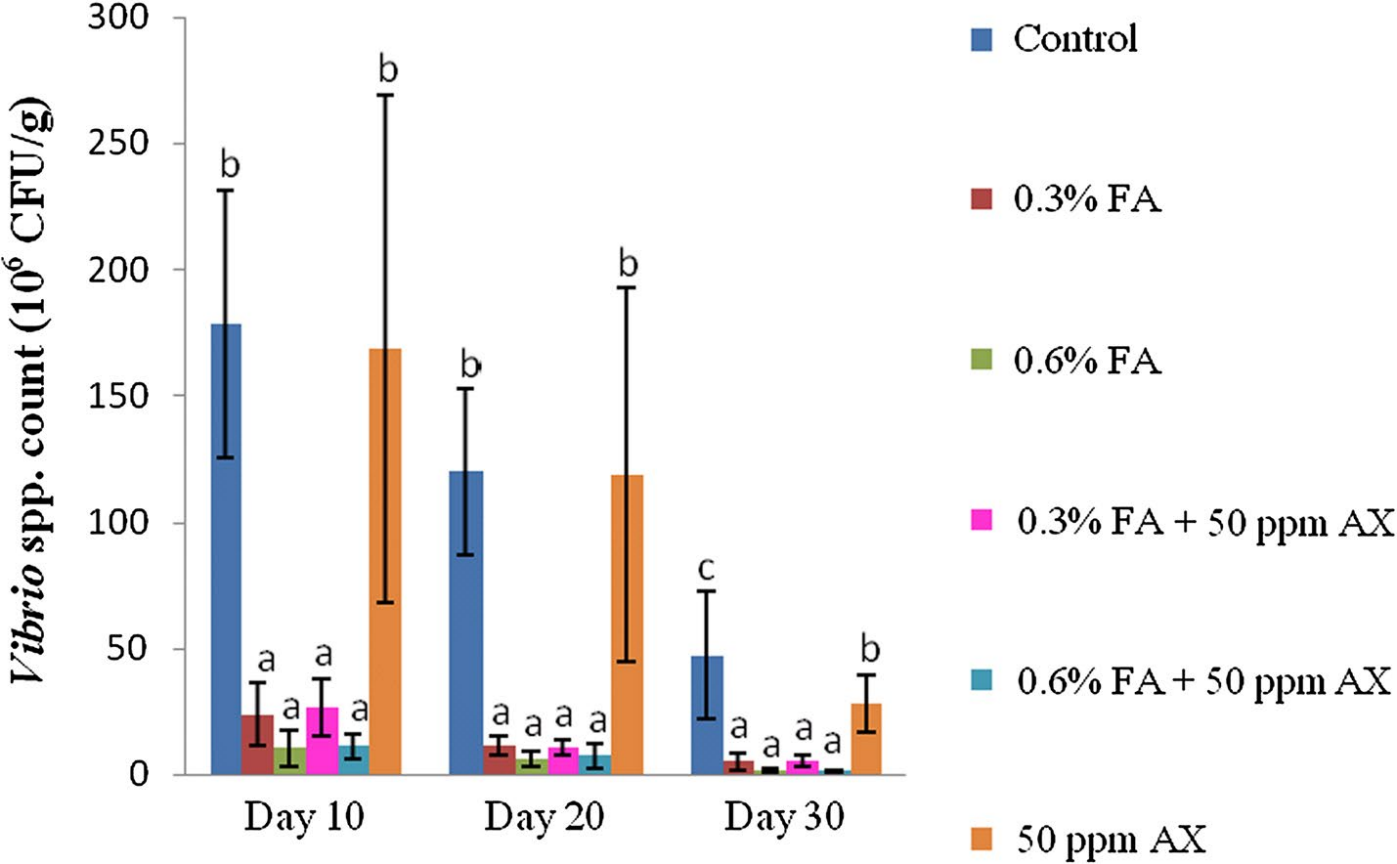
The total number of *Vibrio* spp. (10^6^ CFU/g) in the intestine of Pacific white shrimp (n = 5) after being challenged with *V. parahaemolyticus* at 10^4^ CFU/ml. The data are presented as the mean ± standard deviation. *Different letters* above the *bars* indicate whether means are significantly different from each other (p < 0.05)

**Fig. 2:**
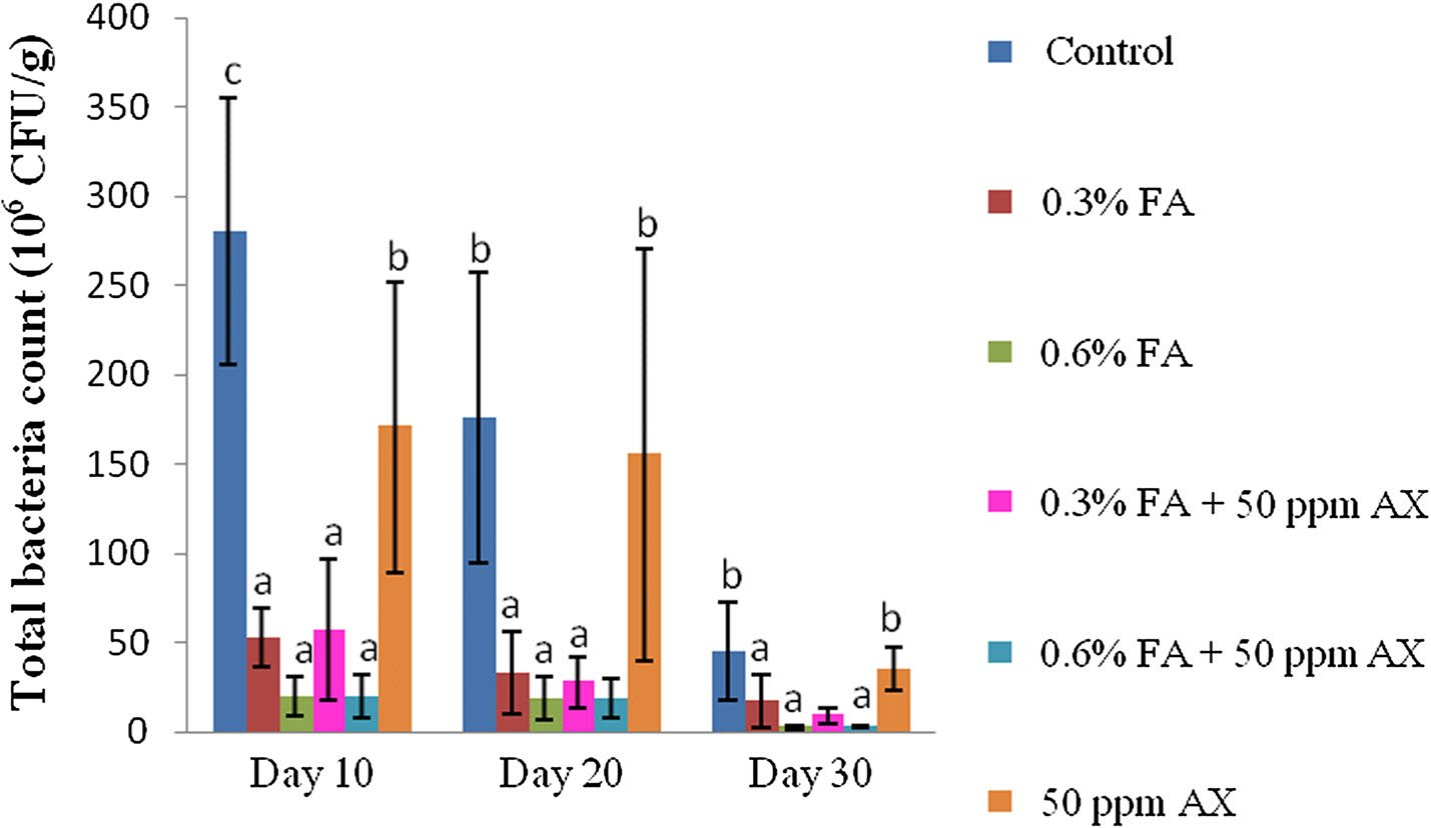
The total number of bacteria (10^6^ CFU/g) in the intestine of Pacific white shrimp (n = 5) after being challenged with *Vibrio parahaemolyticus* at 10^4^ CFU/mL. The data are presented as the mean ± standard deviation. *Different letters* above the *bars* indicate whether means are significantly different from each other (p < 0.05)

For the intestinal bacterial study, both *Vibrio* spp. count and total bacterial count of all four FA-fed groups (namely, 0.3 % FA, 0.6 % FA, 50 ppm AX + 0.3 % FA, and 50 ppm AX + 0.6 % FA) were significantly lower than the control and the 50 ppm AX-fed groups throughout the feeding trial. The lowest intestinal bacterial counts were observed in shrimps with a diet containing the high dose of FA (i.e. 0.6 % FA). On the 30th day of the experiment, the two lowest intestinal *Vibrio* spp. counts were observed in 50 ppm AX + 0.6 % FA and 0.6 % FA groups (1.30 ± 0.58 and 1.60 ± 0.70 × 10^6^ CFU/g, respectively), whereas the highest count was in the control group (47.20 ± 25.40 × 10^6^ CFU/g). Similarly, the two lowest total bacterial counts were in the 0.6 % FA and 50 ppm AX + 0.6 % FA groups (2.80 ± 1.30 and 3.10 ± 0.70 × 10^6^ CFU/g, respectively), while the highest count was from the control group (45.00 ± 27.40 × 10^6^ CFU/g) (Figs. 1, 2).

**Fig. 3:**
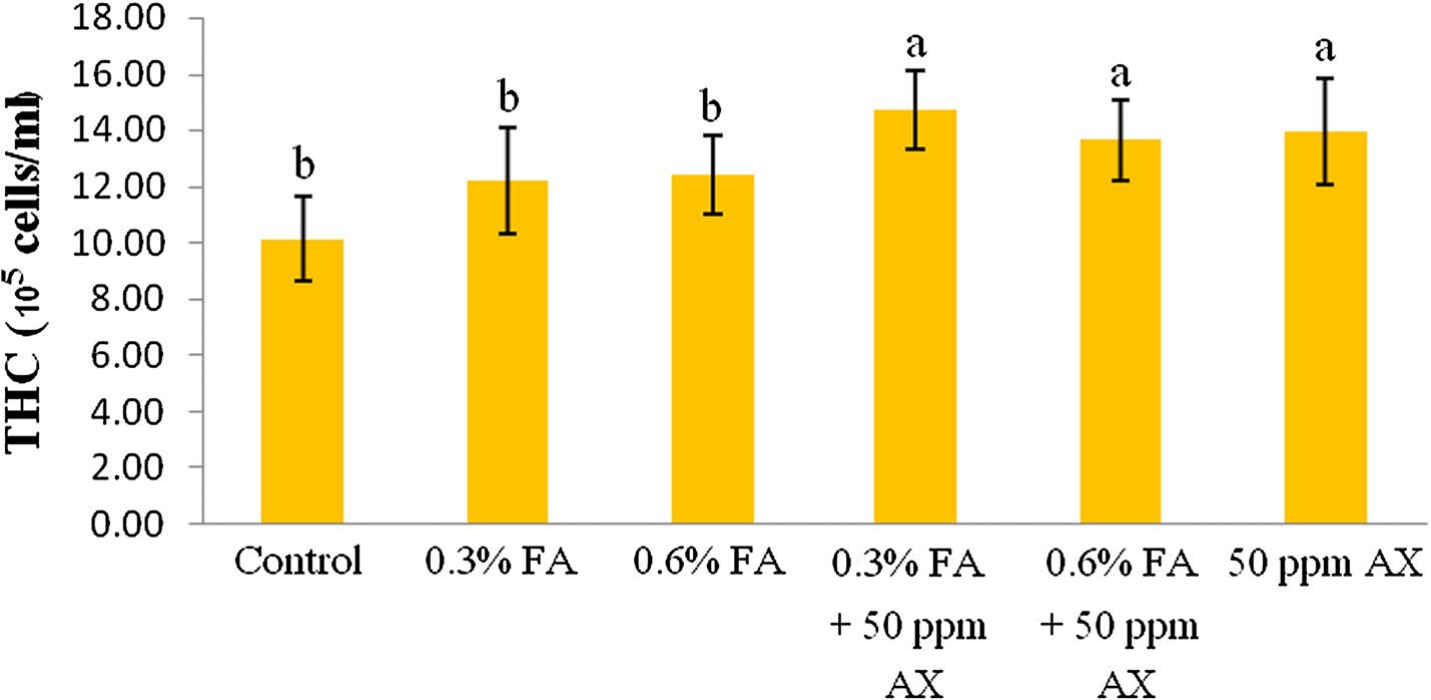
The total hemocyte count (10^5^ cells/ml) of Pacific white shrimp (n = 10) after being challenged with *Vibrio parahaemolyticus* at 10^4^ CFU/mL. The data are presented as the mean ± standard deviation. *Different letters* above the *bars* indicate whether means are significantly different from each other (p < 0.05)

**Fig. 4:**
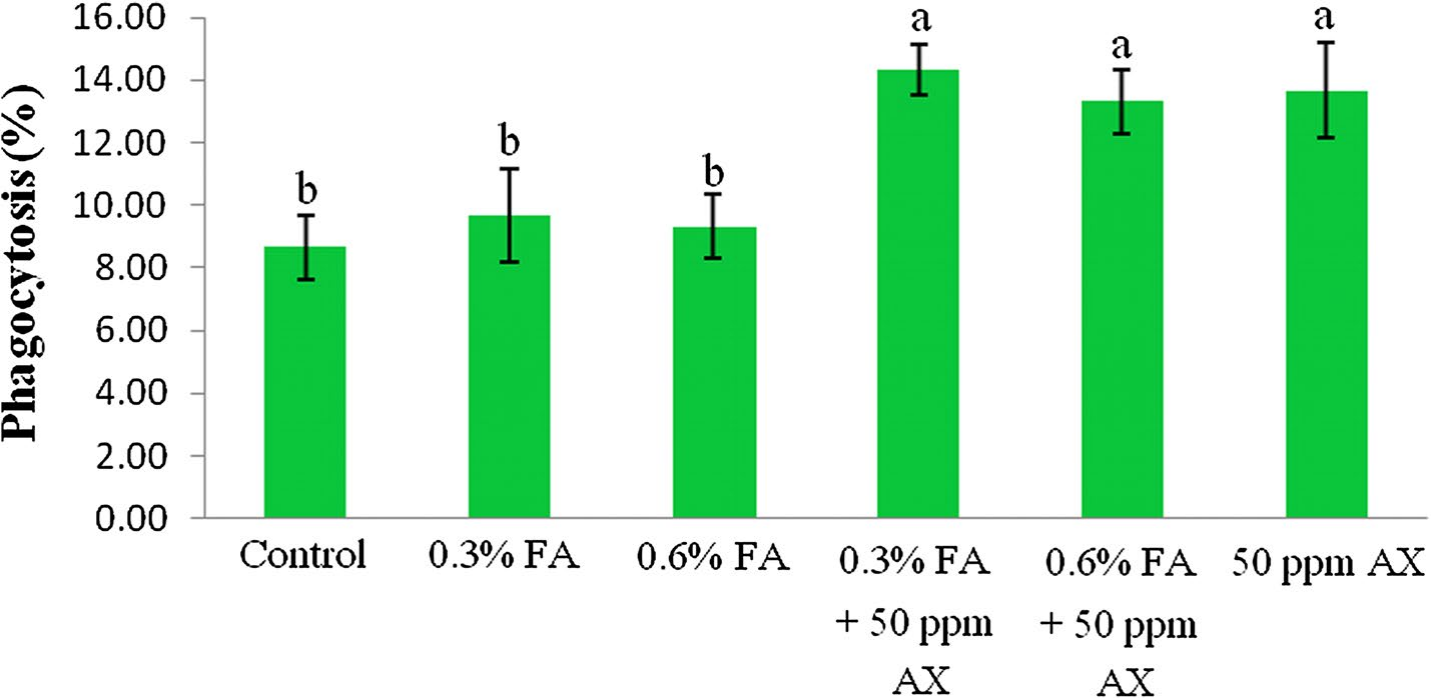
The phagocytosis activity (%) of Pacific white shrimp (n = 10) after being challenged with *Vibrio parahaemolyticus* at 10^4^ CFU/mL. The data are presented as the mean ± standard deviation. *Different letters* above the *bars* indicate whether means are significantly different from each other (p < 0.05)

**Fig. 5:**
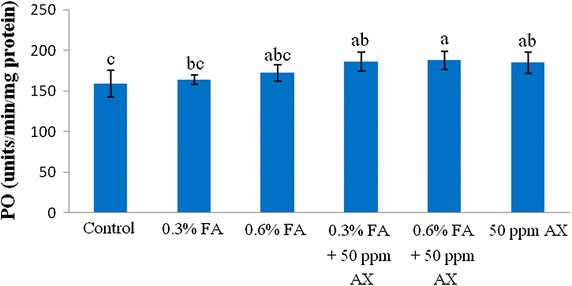
Phenoloxidase activity (units/min/mg protein) of Pacific white shrimp (n = 10) after being challenged with *Vibrio parahaemolyticus* at 10^4^ CFU/ml. The data are presented as the mean ± standard deviation. *Different letters* above the *bars* indicate whether means are significantly different from each other (p < 0.05)

**Fig. 6:**
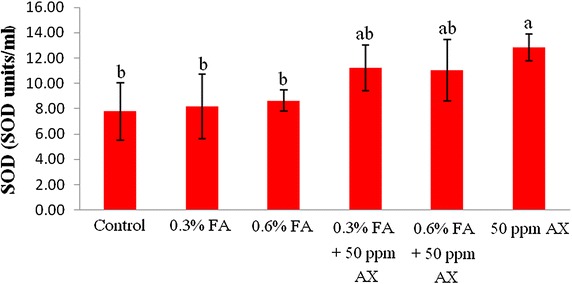
Superoxide dismutase activity (SOD units/mL) of Pacific white shrimp (n = 10) after being challenged with *Vibrio parahaemolyticus* at 10^4^ CFU/ml. The data are presented as the mean ± standard deviation. *Different letters* above the *bars* indicate whether means are significantly different from each other (p < 0.05)

The immune parameters of shrimp were significantly influenced by AX in the shrimp feed. Shrimps fed with diets containing AX (namely, 50 ppm AX + 0.3 % FA, 50 ppm AX + 0.6 % FA, and 50 ppm AX) had a total hemocyte count (THC) (Fig. 3), phagocytosis activity (Fig. 4), and phenoloxidase (PO) activity (Fig. 5) significantly higher than the control and the FA groups. Superoxide dismutase (SOD) activity (Fig. 6) of 50 ppm AX-fed shrimps but not the 50 ppm AX + 0.3 % FA and 50 ppm AX + 0.6 % FA groups showed a significant increase compared with shrimps that not fed AX. However, the bactericidal activity of the shrimp’s hemolymph from all groups was at the same serum dilution, being 1:4 (Additional file 1: Table S3).

## Discussion

Organic acids are widely used as animal food additives and preservatives for preventing food deterioration. As a group these compounds primarily include the saturated straight-chain monocarboxylic acids and their derivatives (Ricke 2003). Many of them are available as sodium, potassium, or calcium salts because they are generally odorless, easier to handle, less corrosive, and may have higher solubility than free acids (Papatsiros and Billinis 2012). Organic acids possess antimicrobial activity against several pathogenic bacteria such as *Escherichia coli*, *Salmonella* spp., and *Vibrio* spp. (Ricke 2003; Papatsiros and Billinis 2012; da Silva et al. 2013). Undissociated forms of organic acids can easily penetrate bacterial cell membranes, and dissociate into anions and H^+^ within the cytoplasm (Ricke 2003; Beales 2004; Lückstädt and Mellor 2011). Once inside the bacterial cells, they reduce intracellular pH and disrupt the cytoplasmic membrane, protein synthesis system, genetic materials, and metabolic enzymes. In addition, because the bacterial cell uses ATP to pump the excess H^+^ out of cells, organic acids also deplete ATP levels and affect the cell’s ability to maintain pH homeostasis (Ricke 2003; Beales 2004; Lückstädt and Mellor 2011). However, not all organic acids have effects on bacteria. In fact, organic acids associated with specific antimicrobial activity are short-chain acids (C1–C7) and are either simple monocarboxylic acids such as formic, acetic, propionic, and butyric acid, or are carboxylic acid bearing a hydroxyl group such as lactic, malic, tartaric, and citric acids (Dibner and Buttin 2002; Papatsiros and Billinis 2012).

Organic acids are mainly used as feed additives for improving growth performance of pigs and poultry (Dibner and Buttin 2002; Franco et al. 2005; Lückstädt and Mellor 2011; Papatsiros and Billinis 2012); there are also reports on the benefit of organic acids in aquatic animals, including red hybrid tilapia (Ng et al. 2009; Koh et al. 2014), yellowtail (Sarker et al. 2012), sturgeon (Khajepour and Hosseini 2012), rohu (Baruah et al. 2007), black tiger shrimp (Ng et al. 2015), and Pacific white shrimp (Walla et al. 2012; da Silva et al. 2013; Su et al. 2014; Romano et al. 2015). Nevertheless, the result from experiment 1 showed that shrimp fed FA had significantly lower body weight than shrimp fed 50 ppm AX and the growth was slightly less than the control group. This indicated that formic acid did not promote the growth of the shrimp and might have some negative effect on shrimp growth. Other short chain fatty acids may enhance the growth, for example, 2 % organic acids blend (consisted of a blend of formic, lactic, malic and citric acids) (Romano et al. 2015) or 2 g/kg citric acid (Su et al. 2014).

Despite no clear improvement of growth and survival of uninfected shrimp postlarvae in our study, the use of formic acid did increase the survival rate of *V. parahaemolyticus*-infected juvenile shrimps significantly compared with the control group. This result was consistent with the intestinal bacterial study, i.e. shrimp fed formic acid had significantly lower *Vibrio* spp. and total bacterial counts compared with those fed no formic acid. The similarity between *Vibrio* spp. counts and total bacterial counts suggested that *Vibrio* spp. are significant component of shrimp’s intestinal microflora (Moss et al. 2000; Oxley et al. 2002; Liu et al. 2011). The antimicrobial effect of formic acid against *Vibrio* spp. was reported in vitro as well (Mine and Boopathy 2011; Adams and Boopathy 2013; da Silva et al. 2013). Considering all of these aspects, the antibacterial property of formic acid may reduce *Vibrio* infection to Pacific white shrimp (Papatsiros and Billinis 2012; Adams and Boopathy 2013; da Silva et al. 2013) by penetrating the cell wall of bacteria in the undissociated form, then releasing H^+^ and destabilizing the intracellular pH of the bacterial cytoplasm, leading to death (da Silva et al. 2013).

Astaxanthin is a pigment that belongs to the xanthophyll class (the oxygenated derivatives of carotenoids) and widely used in salmon and crustacean aquaculture to provide a desirable reddish-orange color. Astaxanthin possesses a potent antioxidant property and has an important role in larval growth and reproductive success of crustaceans. It occurs naturally in green microalgae *Haematococcus pluvialis* and red yeast *Xanthophyllomyces dendrorhous* (*Phaffia rhodozyma*). However, since farmed crustaceans often do not have the opportunity to access a natural source of astaxanthin, and they cannot synthesize carotenoids *de novo*, total astaxanthin must be obtained from their feed (Higuera-Ciapara et al. 2006; Seabra and Pedrosa 2010).

The growth of AX-fed shrimp in the experiment 1 was significantly better than the FA-fed shrimp, but was not significantly different from the control group. The survival rate of shrimp fed with AX was not significantly different from the control group. However, the survival rate of *V. parahaemolyticus*-infected shrimp in the experiment 2 was improved significantly. Nevertheless, unlike formic acid, astaxanthin did not suppress intestinal bacterial populations, suggesting that other mechanisms may be responsible for the increased survival rate. In fact, many immune parameters of astaxanthin-fed shrimps were improved, including total hemocyte count (THC), phagocytosis activity, phenoloxidase (PO) activity, and superoxide dismutase (SOD) activity. These outcomes suggest that astaxanthin had an immunostimulatory property preventing *V. parahaemolyticus* infection in Pacific white shrimp. Antioxidant activity of carotenoids may be involved in the immunomodulatory effect; by quenching singlet oxygen and free radicals, carotenoids can protect white blood cells from oxidative damage (Bendich 1989). Superoxide dismutase (SOD) is an antioxidant enzyme that protect cells against oxidative stress by scavenges superoxide anion (O_2_^−^) and it is used as an indicator of immune responses (Campa-Córdova et al. 2002a, 2002b). Given that astaxanthin also possesses an antioxidant property, this is suggests that such mechanism must be take part in immunomodulation. Furthermore, the effects of carotenoids on enhancing cell-mediated and humoral immune responses of vertebrates are also documented (Bendich 1989; Chew and Park 2004). Several studies have reported that dietary carotenoids can increase the immune parameters, enhance the survival rate, or act as a prophylactics to pathogens for many aquatic animals such as common carp (Anbazahan et al. 2014; Sowmya and Sachindra 2015), rainbow trout (Amar et al. 2001), Pacific white shrimp (Flores et al. 2007; Niu et al. 2009), black tiger shrimp (Supamattaya et al. 2005), kuruma prawn (Chien and Shiau 2005), and giant freshwater prawn (Angeles et al. 2009). Our results are somewhat similar to these studies.

Even if formic acid and astaxanthin have different modes of action to shrimp, both had positive effects on their resistance to bacterial challenge. Meanwhile, our results showed that a combination of formic acid and astaxanthin was no better than using singly or in combination. The only exception was that uninfected shrimp fed 0.6 % FA + 50 ppm AX had a significantly higher survival rate compared with the control group. In general, formic acid (FA 0.3 and 0.6 %) and astaxanthin (50 ppm AX) were equally effective in preventing *V. parahaemolyticus* infection of Pacific white shrimp. Given the fact that formic acid is less expensive than astaxanthin, using formic acid as feed additive in shrimp farming may be considered more economically worthwhile.

## Conclusion

Astaxanthin (50 ppm AX) can be used as growth promoter in uninfected Pacific white shrimp, while formic acid (0.3 and 0.6 % FA) and AX can enhance the survival rate of *Vibrio parahaemolyticus*-infected shrimp in laboratory conditions. In addition, FA-fed shrimps had lower intestinal *Vibrio* spp. and total bacterial counts, whereas AX-fed shrimps showed improvement in many immune parameters. The results of our study suggest that using FA, AX, and their combination as a feed additive can prevent *V. parahaemolyticus* infection in shrimps.

## Additional file

**Additional file 1.** **Table S1**. The body weight and survival rate of Pacific white shrimp after 60 day of dietary administration. **Table S2**. The body weight and survival rate of Pacific white shrimp at 30 day after being challenged with *Vibrio parahaemolyticus* 10^4^ CFU/ml. **Table S3**. The bactericidal activity of the shrimp’s hemolymph from all groups.
