# Supplementary material for: Effect of dietary formic acid and astaxanthin on the survival and growth of Pacific white shrimp (Litopenaeus vannamei) and their resistance to Vibrio parahaemolyticus
Source: Springerplus. 2015 Aug 21;4:440. doi: 10.1186/s40064-015-1234-x (PMC4545949; doi:10.1186/s40064-015-1234-x)
Supplement: Supplementary file 1 — Additional file 1. Table S1. The body weight and survival rate of Pacific white shrimp after 60 day of dietary administration. Table S2. The body weight and survival rate of Pacific white shrimp at 30 day after being challenged with Vibrio parahaemolyticus 104 CFU/ml. Table S3. The bactericidal activity of the shrimp’s hemolymph from all groups. [file 40064_2015_1234_MOESM1_ESM.doc]

**Table 1** **Body weight and survival rate of Pacific white shrimp after 60 days of feeding with six different diets**

| Treatment groups | Body weight (g) | Survival rate (%) |
| --- | --- | --- |
| Control | 4.18 ± 0.05^abc^ | 64.33 ± 10.12^b^ |
| 0.3 % FA | 3.68 ± 0.49^c^ | 72.33 ± 9.94^ab^ |
| 0.6 % FA | 3.88 ± 0.22b^c^ | 67.67 ± 14.29^ab^ |
| 0.3 % FA + 50 ppm AX | 4.38 ± 0.37^ab^ | 78.67 ± 7.77^ab^ |
| 0.6 % FA + 50 ppm AX | 4.05 ± 0.21^abc^ | 82.33 ± 8.32^a^ |
| 50 ppm AX | 4.45 ± 0.45^a^ | 78.33 ± 9.26^ab^ |

The data are presented as the mean ± standard deviation. Means in the same column with different superscripts are significantly different from each other (p<0.05).

**Table 2 Weight gain and survival rate of Pacific white shrimp fed with six different diets for 30 days after being challenged with *Vibrio parahaemolyticus* at 10^4^ CFU/ml**

| Treatment groups | Weight gain (g) | Survival rate (%) |
| --- | --- | --- |
| Control | 2.73 ± 1.77^a^ | 20.00 ± 17.32^c^ |
| 0.3 % FA | 2.13 ± 0.74^a^ | 45.83 ± 16.78^b^ |
| 0.6 % FA | 1.82 ± 0.82^a^ | 65.00 ± 5.77^a^ |
| 0.3 % FA + 50 ppm AX | 2.97 ± 0.83^a^ | 54.17 ± 5.09^ab^ |
| 0.6 % FA + 50 ppm AX | 2.01 ± 0.69^a^ | 67.50 ± 3.33^a^ |
| 50 ppm AX | 2.39 ± 0.23^a^ | 56.67 ± 17.11^ab^ |

The data are presented as the mean ± standard deviation. Means in the same column with different superscripts are significantly different from each other (p<0.05).

**Table 3 The bactericidal activity of Pacific white shrimp (n = 10) after being challenged with *Vibrio parahaemolyticus* at 10^4^ CFU/ml**

| Treatment groups | Bactericidal activity |
| --- | --- |
| Control | 1:4 |
| 0.3 % FA | 1:4 |
| 0.6 % FA | 1:4 |
| 0.3 % FA + 50 ppm AX | 1:4 |
| 0.6 % FA + 50 ppm AX | 1:4 |
| 50 ppm AX | 1:4 |
